# Metabolomic profiles of intact tissues reflect clinically relevant prostate cancer subtypes

**DOI:** 10.1186/s12967-023-04747-7

**Published:** 2023-11-27

**Authors:** Ilona Dudka, Kristina Lundquist, Pernilla Wikström, Anders Bergh, Gerhard Gröbner

**Affiliations:** 1 Department of Chemistry, Umeå University, Umeå, Sweden; 2 Department of Medical Biosciences, Pathology, Umeå University, Umeå, Sweden

**Keywords:** Metabolomics, Prostate cancer, Subtype, HR MAS NMR, Biomarker

## Abstract

**Background:**

Prostate cancer (PC) is a heterogenous multifocal disease ranging from indolent to lethal states. For improved treatment-stratification, reliable approaches are needed to faithfully differentiate between high- and low-risk tumors and to predict therapy response at diagnosis.

**Methods:**

A metabolomic approach based on high resolution magic angle spinning nuclear magnetic resonance (HR MAS NMR) analysis was applied on intact biopsies samples (n = 111) obtained from patients (n = 31) treated by prostatectomy, and combined with advanced multi- and univariate statistical analysis methods to identify metabolomic profiles reflecting tumor differentiation (Gleason scores and the International Society of Urological Pathology (ISUP) grade) and subtypes based on tumor immunoreactivity for Ki67 (cell proliferation) and prostate specific antigen (PSA, marker for androgen receptor activity).

**Results:**

Validated metabolic profiles were obtained that clearly distinguished cancer tissues from benign prostate tissues. Subsequently, metabolic signatures were identified that further divided cancer tissues into two clinically relevant groups, namely ISUP Grade 2 (n = 29) and ISUP Grade 3 (n = 17) tumors. Furthermore, metabolic profiles associated with different tumor subtypes were identified. Tumors with low Ki67 and high PSA (subtype A, n = 21) displayed metabolite patterns significantly different from tumors with high Ki67 and low PSA (subtype B, n = 28). In total, seven metabolites; choline, peak for combined phosphocholine/glycerophosphocholine metabolites (PC + GPC), glycine, creatine, combined signal of glutamate/glutamine (Glx), taurine and lactate, showed significant alterations between PC subtypes A and B.

**Conclusions:**

The metabolic profiles of intact biopsies obtained by our non-invasive HR MAS NMR approach together with advanced chemometric tools reliably identified PC and specifically differentiated highly aggressive tumors from less aggressive ones. Thus, this approach has proven the potential of exploiting cancer-specific metabolites in clinical settings for obtaining personalized treatment strategies in PC.

**Supplementary Information:**

The online version contains supplementary material available at 10.1186/s12967-023-04747-7.

## Background

One of the most challenging aspects in current prostate cancer (PC) diagnosis and therapy is the unambiguous and correct classification of patients according to tumor aggressiveness and molecular subtype [1]. For a long time, prostate cancer has been recognized as a heterogenous disease ranging from indolent asymptotic cases to very aggressive, metastatic and lethal forms. However, recently several molecular subtypes of PC have been identified with distinct mutational profiles, transcriptomic profiles, and biological processes of relevance for predicting patient risk at diagnosis and/or outcome after treatment [2–13]. Cuzick et al. [8] used RNA expression signature derived from cell cycle proliferation genes in PC patients for predicting biochemical recurrence after radical prostatectomy. Another genomic classifier based on 22 gene transcripts was developed to predict early metastasis after surgery [9], and a Genomic Prostate Score was established to predict PC aggressiveness based on a panel of 17 genes [10]. By exploiting more comprehensive transcriptome profiles, You et al. [11] were able to classify three distinct subtypes of PC tissues, called PCS1–3. Tumors belonging to PCS1 and PCS2 groups reflected luminal subtypes, while PCS3 represented a basal subtype. A similar classification was obtained based on the PAM50 transcriptomic panel, originally developed for classification of breast tumors and now used by Zhao et al. [12] in PC to predict prognosis after androgen deprivation therapy. Additionally, a large meta-analysis of gene expression profiles from seven cohorts enabled the differentiation of tumors into 4 subtypes that were directly correlated to tumor aggressiveness and susceptibility to treatments [13].

Recent transcriptomic studies by us identified three clinically relevant subtypes of PC bone metastases: MetA, MetB and MetC, of which MetB shows a particularly aggressive behavior [6, 7]. The main features of the MetA-C subtypes are comparable to those of the PCS1-3 subtypes [11] and the luminal A, B and basal subtypes described for primary tumors [12]. The MetB subtype is similar to the PCS1/luminal B groups, showing high cell proliferation, poor differentiation and prognosis. MetA reflects the PCS2/luminal A groups as being hormone-sensitive and less aggressive, and the MetC is similar to the PCS3/basal-cell-like cancers. Identification of the aggressive MetB subtype was successfully achieved either by analysis of 157 MetA-C-differentiating transcripts or by a combination of two immunohistochemical markers [14]. There, Ki67 was used as a surrogate marker for tumor cell proliferation and prostate specific antigen (PSA) as marker for tumor cell differentiation and androgen dependency. Importantly, the Ki67/PSA immunoreactivity score of diagnostic tumor biopsies seems to allow prediction of patient prognosis and bone metastatic subtype [14, 15]. Together, those various subtypes provide valuable insights into prostate tumor heterogeneity and mechanisms involved in tumor progression [16, 17], but validated classifiers are still absent in the clinical environment to enable tailored subtype-specific therapies. Moreover, there is also only scarce knowledge about the underlying molecular mechanisms and biochemical pathways driving various PC subtypes.

A promising strategy in the hunt for mechanistic insights, potent biomarkers and therapeutic targets to improve diagnosis, prognosis and therapy of PC is exploiting metabolomic approaches on intact prostate tumor tissues [18–21]. Using intact tissue biopsy samples is vital in identifying biomarkers which originate at the direct tissue location and therefore directly reflect ongoing cancer pathogenesis, including aberrant molecular biochemical processes and regulation [22]. To provide the metabolomic information and identify crucial biomarkers in intact tissue biopsies in a non-destructive way, high resolution magic angle spinning nuclear magnetic resonance (HR MAS NMR) has emerged as a powerful technique in recent years [23, 24]. Despite being less sensitive than mass spectroscopy (MS), this technique has been successfully applied in metabolomics, especially in cancer related studies [21, 25, 26]. The HR MAS NMR approach requires only minimal sample preparation and the samples are available afterwards for subsequent analysis by histopathology, gene expression profiles and other methods. Most importantly, NMR provides highly reproducible, quantitative metabolomic profiles and keeps tissue architectures preserved and degradation at minimum since NMR spectra are acquired at low temperatures. Therefore, the HR MAS NMR technique on intact biopsies is ideal to identify specific correlations between metabolites and pathological parameters upon taking in account the fractions of cancerous and other cells in the tissue [21, 25]. Using this approach, we have recently studied PC heterogeneity and identified significant metabolomic differences between *TMPRSS2-ERG-*positive and -negative PC cases [23].

In this study, HR MAS NMR on intact prostate tumor biopsies provided molecular information for PC tissues reflecting key biochemical processes and their varying regulation not only as a function of tumor differentiation (according to Gleason scores and the International Society of Urological Pathology (ISUP) grading system [27]), but also reflecting three previously established PC subtypes, differentiated based on Ki67/PSA immunoreactivity; the “subtype A” showing low Ki67, high PSA immunoreactivity and favorable prognosis, the “subtype B” characterized by high Ki67, low PSA immunoreactivity and poor prognosis, and the “non-AB subtype” having features between both other two subtypes [14]. Thus, our results show also the huge potential for detecting PC and particular aggressive tumors by non-invasive NMR on intact biopsies, and provide opportunities for precision medicine and tailor-made treatment of patients based on specific PC subtypes.

## Material and methods

### Patients and tissue samples

This study was conducted in accordance with the Declaration of Helsinki, and the study protocol was approved by the research ethical committee at Umeå University hospital (Regional Ethical Review Board in Umeå). Written informed consent was obtained from each patient. Fresh-frozen prostate tissues were obtained from a total of 31 patients treated by prostatectomy at the Urology Clinic, Umeå University Hospital, between 2009 and 2012. Patient ages ranged between 58 and 74 years and preoperative serum PSA levels between 2.8 and 28 µg/L. No prostate cancer treatment had been given prior to surgery. Immediately after surgical removal the prostates were brought to the Pathology Department and cut in 0.5 cm thick slices. From each prostate 20 samples were punched from the slides using a 0.5 cm steel cylinder and frozen in – 70 ºC within 30 min after surgery. The prostate slices were then fixed in 4% formaldehyde for 24 h, dehydrated, embedded in paraffin (FFPE), cut in 5 µm thick sections and stained with hematoxylin–eosin (H&E). The different frozen samples could thus be identified as individual holes in the paraffin sections.

Finally, prostate tumor tissues and adjacent control tissues without morphological changes were used from 28 patients, and solely benign samples were available from three patients (their frozen samples did not contain tumor). From 13 patients with multifocal cancer, multiple PC samples were collected (2–3) and from 12 patients multiple benign samples (2–5) were collected. In total, 92 prostate tissue samples (43 cancer and 49 benign) were obtained. Some samples were cut into 2–3 replicates, to fit inserts used for sample rotors required for NMR experiments. Due to the observed heterogeneity those replicates were treated as individual samples. Altogether, 111 samples (53 benign and 58 cancer) were assigned to metabolomic analysis. Gleason grade, ISUP grade and the percentage area representing cancerous tissue were estimated in the samples. The clinicopathological characteristics of patients and samples are summarized in Table 1.

**Table 1:** Patients and samples characteristics

	All	PC subtype A	PC subtype B	PC subtype non-AB
Patients	31 (31)^a^	11 (11)^a^	15 (15)^a^	5 (5)^a^
Benign samples	53 (48)^a^			
Malignant samples	58 (56)^a^	22 (21)^a^	29 (28)^a^	7 (7)^a^
Percentage of malignancy				
% ≤ 10	5	3	1	1
10 < % ≤ 25	16	10	3	3
25 < % ≤ 50	9 (7)^a^	5 (4)^a^	3 (2)^a^	1
50 < % ≤ 75	9	0	9	0
75 < % ≤ 100	19	4	13	2
ISUP grade group/Gleason score				
1/3 + 3	4	4	0	0
2/3 + 4	31 (29)^a^	15 (14)^a^	13 (12)^a^	3
3/4 + 3	17	3	10	4
4/4 + 4	6	0	6	0

^a^Numbers in brackets indicate number of samples included in multivariate analysis after removing samples with high lipid content; ISUP grade according to the International Society of Urological Pathology

### A combinatory Ki67/PSA immunoreactivity score

Tumor samples were stained for PSA and Ki67, as described previously [14]. The Ki67 labelling index was determined by counting at least 500 tumor cells situated in ten randomly selected areas within the tumor. The PSA staining index in the tumor was measured by multiplying staining intensity (graded from high = 3, moderate = 2, low = 1, and absent = 0) by distribution (1 = 0–25%, 2 = 26–50%, 3 = 51–75%, 4 > 75%), giving a score ranging from 12 (high intensity in most cells as in normal prostate glands) to 0 (no staining). By using the combinatory PSA and Ki67 staining data, patients were categorized into 3 different subtypes of PC using cut-offs as defined in our previous paper [14]: subtype A with low Ki67 (Ki67 ≤ 3%) and high PSA (> 8); subtype B with high Ki67 (> 3%) and low PSA (< 8); subtype non-AB, samples not classified as A or B.

### ^1^H HR MAS NMR on intact tissue biopsies

^1^H HR MAS NMR-based metabolomics analysis was performed on intact tissue samples at a 500 MHz NMR spectrometer (Bruker Biospin, GmbH, Germany), as described recently by us [23]. Shortly, all tissue samples were cut to fit a disposable 30 μL insert and kept on ice at all times during the preparation process. Inserts were transferred into 4 mm zirconia MAS rotors and spun at 5 kHz at 277 K to prevent tissue degradation. A Carr-Purcell-Meiboom-Gill (CPMG) NMR pulse sequence was applied with a spectral width of 20 ppm, 1024 scans, echo time of 0.2 ms, total acquisition time of 1.64 s, recycle delay of 1.5 s and 32 K data points. All NMR spectra were manually corrected using TopSpin 3.6.5 software (Bruker Biospin, GmbH, Germany). Seven NMR spectra were excluded from further analysis due to visible very high lipid content in those samples. The NMR spectra were imported into MATLAB 2017a (The Mathworks, Inc., USA) and aligned using icoshift 1.2. Manual integration of the NMR peaks was performed to a linear baseline on all spectra in parallel using an in-house developed MATLAB R2017a routine as used before [28]. The integrated data were normalized with respect to the total sum of the spectrum. Finally, the main metabolite identification was carried out using the Chenomx NMR suite professional (version 8.6, Chenomx Inc., Edmonton, Canada).

### Multivariate analysis

The NMR derived spectral dataset was further analyzed by multivariate analysis methods as provided by SIMCA V17 (Umetrics, Umeå, Sweden). Since metabolomic data, especially NMR spectral data, are characterized by a high degree of collinearity, we applied multivariate analysis methods, the principal component analysis (PCA) and the orthogonal partial least squares discriminant analysis (OPLS-DA). Those methods take into account correlations between metabolites and have been widely used to identify biomarkers in metabolomics studies [29, 30]. PCA was used to generate a first overview of information contained in the data, since it reduces the dimensionality of such datasets to increase interpretability and to minimize information loss. Thus, the original data can be described in a lower-dimensional space, defined by the principal components, which are ordered according to their ability to capture the total variance of the data. The score values represent the coordinates of the samples in the lower-dimensional space defined by the principal components. The principal components are displayed in a two-dimensional score plot, allowing visualization of the distribution and grouping of the samples in the new variable space [29]. Accordingly, by inspecting the score plot the homogeneity of the samples can be evaluated and any possible trends and outliers between the samples become visible. Thereafter, a supervised multivariate analysis OPLS-DA was performed to identify the discriminatory features for each comparison of the different assigned groupings. Significant metabolites were selected based on the p(corr) > 0.5 from the OPLS-DA models, where p(corr) is defined as the loadings rescaled as a correlation coefficient between the original data and the scores, thereby standardizing the range from − 1.0 to 1.0. There is no consensus on what p(corr) cutoff represents significance, but an absolute p(corr) > 0.4–0.5 is commonly used [31–33]. The quality of the OPLS-DA models was evaluated by using the default sevenfold crossvalidation in SIMCA and the built-in permutation plot (in short: permuting the y-variable 200 times and subsequently correlating these results with that of the original models). Analysis of variance of cross-validated predictive residuals (CV-ANOVA) was used to assess the significance of the OPLS-DA models, where a *p*-value lower than 0.05 is associated with a significant model.

### Univariate statistical analysis

Metabolomic differences among assigned groups were tested by using the ANOVA with post hoc Benjamini-Hochberg (FDR, false discovery rate) correction for multiple comparisons (q < 0.05). Univariate statistical analyses were performed with GraphPad Prism (GraphPad Software Inc., San Diego, CA, USA) version 9.4.1. Correlation between metabolite levels and Ki67 and PSA values were calculated with Pearson correlation (two-tailed *p*-value, 95% confidence interval) in GraphPad Prism. Additionally, to consider effect of age and serum PSA levels on metabolomic data, we also run Pearson correlation analysis between those two parameters and metabolite levels.

## Results

### Metabolic profiling of clinical prostate tissue samples

Tumor tissues and adjacent benign tissues were available from the majority of cases (n = 28). The stratification of PC tissue samples was carried out with respect to Gleason score, ISUP grade, and specific PC subtypes (see Table 1). From one patient two tumor samples were collected with different Ki67/PSA ratios. Using ^1^H HR MAS NMR-based metabolomics approach we analyzed the metabolic profiles of 111 prostate tissue samples obtained from 31 PC patients treated by radical prostatectomy; an approach described previously [23]. In total, in the main NMR spectral region (0.7–8.5 ^1^H ppm) 39 metabolites could be identified and quantified as summarized in Additional file 1: Table S1.

### Validation: tumor versus adjacent benign prostate tissue

**Fig. 1:**
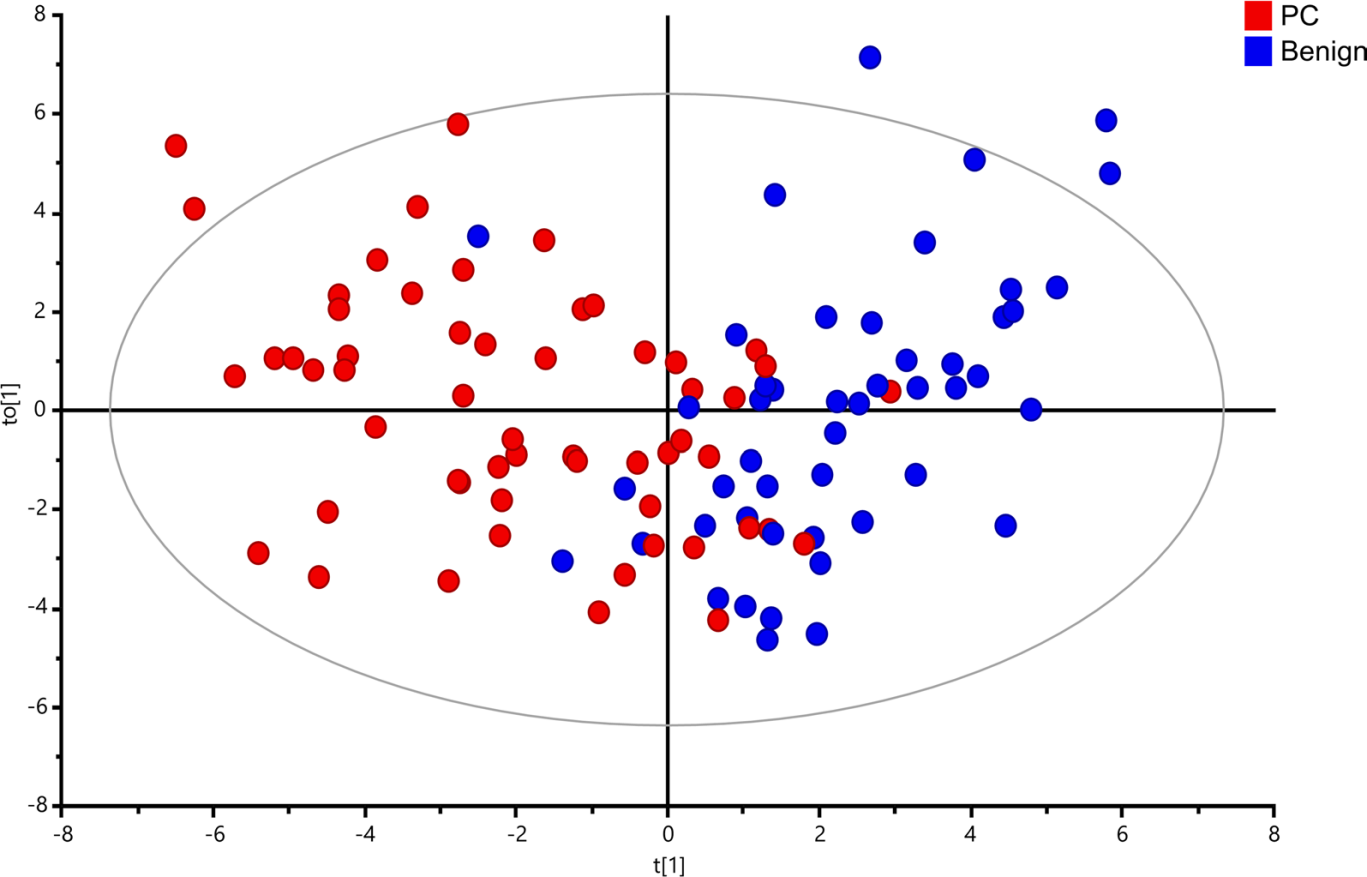
Discrimination between tumor and benign prostate tissues based on metabolic ^1^H HR MAS NMR data. OPLS-DA score plot indicating metabolomics differences between the two groups (blue—benign samples; red—PC samples) with each score representing one subject

**Fig. 2:**
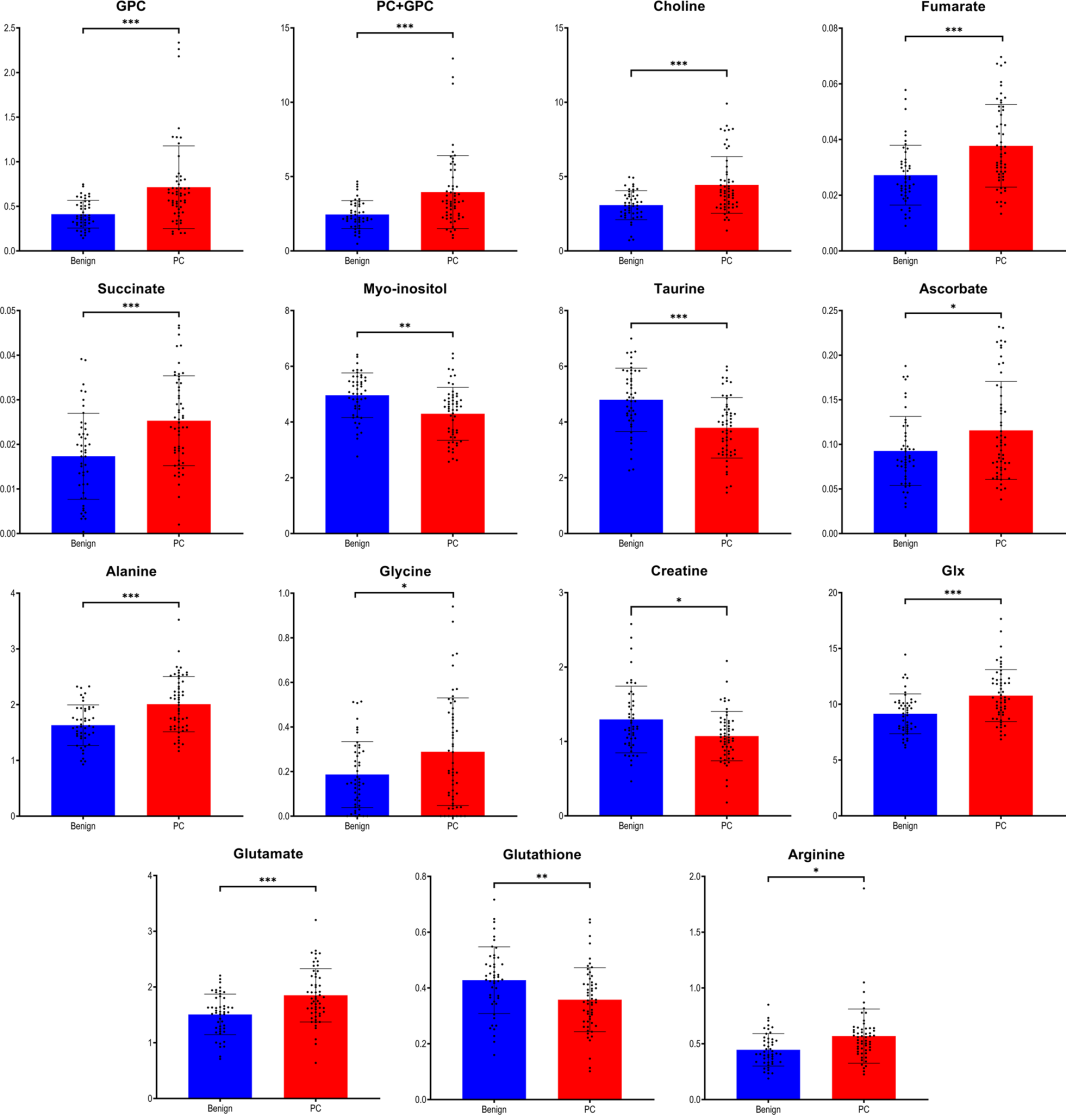
Significant metabolites identified from the benign *vs*. PC (box plots) comparison. Data are expressed as means ± standard deviation and statistical significance was determined using the t-test and the Benjamini–Hochberg adjustment was applied. A *p*-value < 0.05 was considered statistically significant

Multivariate and univariate analysis of metabolomic data is well suited for an unambiguous identification of significant differences in the metabolic profiles between PC and benign prostate tissues. Upon exclusion of samples as outliers (n = 7) due to their very high lipid content based on the PCA plot (Additional file 2: Fig. S1), the metabolic profiles for the remaining samples (48 benign and 56 PC) were subjected to chemometric and univariate statistical analysis. As presented in Fig. 1, the OPLS-DA score plot reveals a significant discrimination between tumor and benign samples with goodness of fit and predictive ability values R2Y and Q2 (0.513 and 0.313, respectively). The discriminant power of the OPLS-DA model was confirmed by random permutation tests as seen in Additional file 3: Fig. S2A and a CV-ANOVA test of the model with *p* = 1.442 × 10^−7^. To identify differential tissue metabolites with high significance their p(corr) values together with their relative concentrations (adjust *p*-values < 0.05) were used. In total, 15 metabolites were selected which display significantly different levels in PC *versus* benign prostate tissues as shown in Fig. 2.

### Metabolite changes with PC aggressiveness

**Fig. 3:**
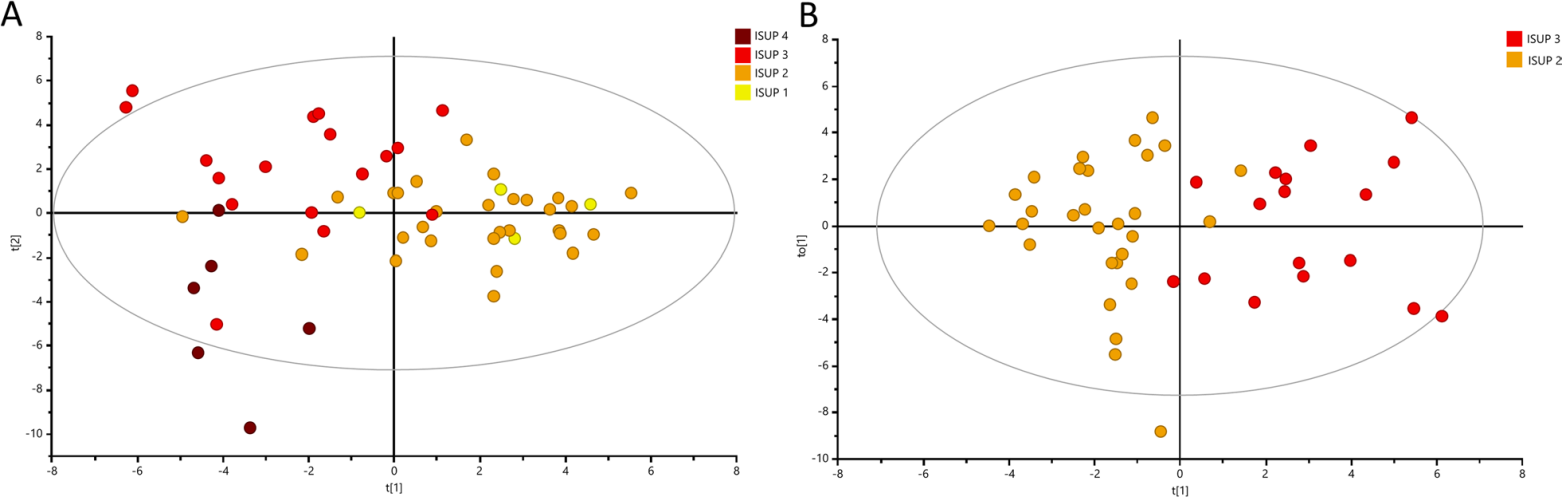
Multivariate analysis obtained from tumor prostate tissues with different aggressiveness assigned based on ISUP Grade Groups. **A** OPLS-DA score plot showing discrimination between four ISUP groups (yellow—ISUP 1; orange—ISUP 2; red—ISUP 3; brown—ISUP 4). **B** OPLS-DA score plot showing discrimination between two ISUP groups (orange—ISUP 2 and brown—ISUP 3)

**Fig. 4:**
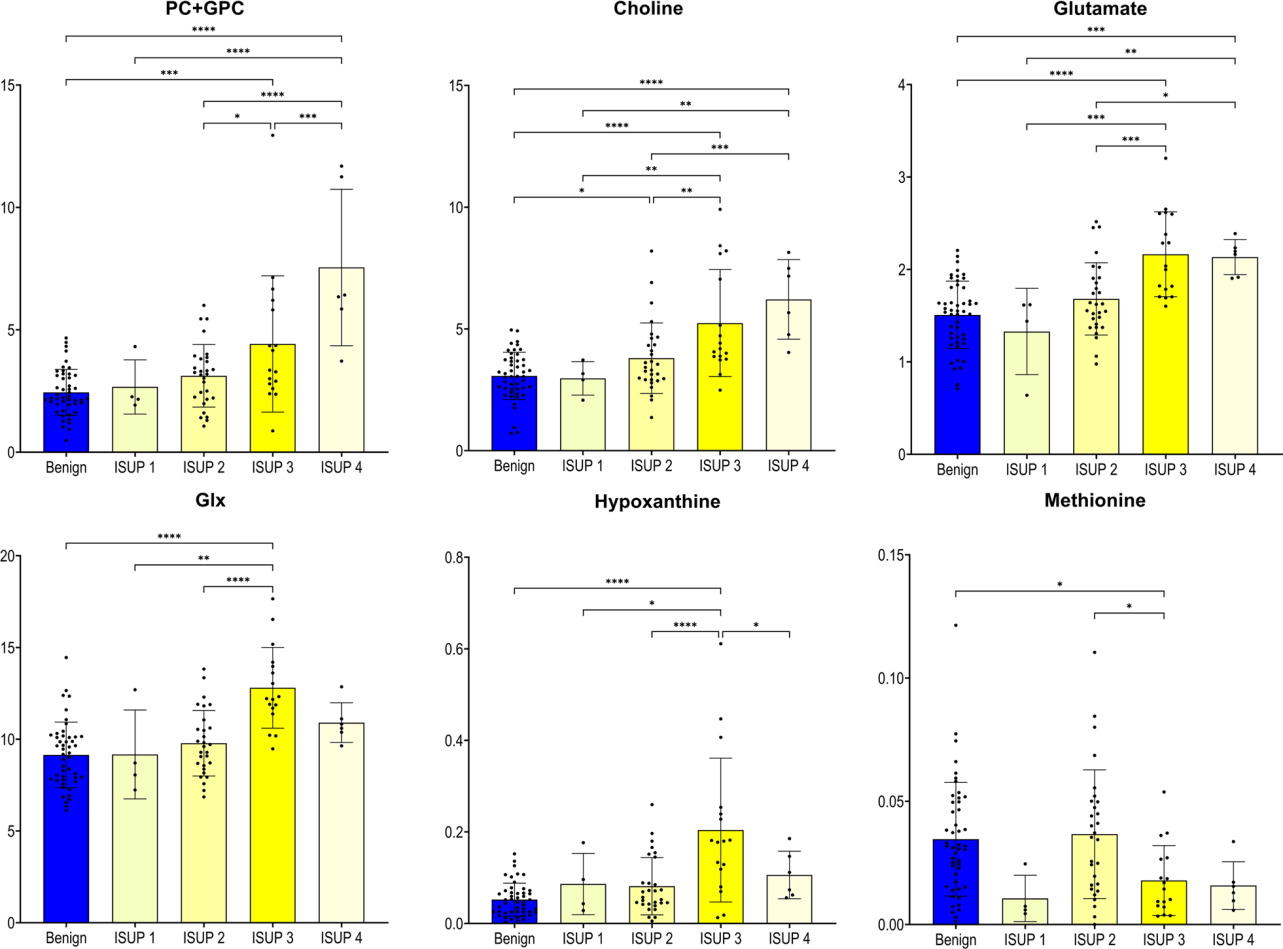
Tissue metabolites as biomarkers to differentiate less-aggressive PC (ISUP 2) from more-aggressive PC (ISUP 3). Box plots showing relative abundances for the six metabolite-panel distinguishing less-aggressive PC (ISUP 2) from more-aggressive PC (ISUP 3) in addition with data from benign prostate tissues and PC with ISUP 1 and PC with ISUP 4. Data are expressed as means ± standard deviation and statistical significance was determined using the one-way ANOVA followed by post-hoc Benjamini-Hochberg (FDR, false discovery rate) test and a *p*-value < 0.05 was considered statistically significant. PC + GPC—peak for combined phosphocholine/glycerophosphocholine metabolites; Glx: combined signal of glutamate/glutamine

To classify the PC samples into different groups based on relative aggressiveness, we initially built an OPLS-DA model including all four ISUP Grade Groups (R2Y = 0.350; Q2 = 0.182; *p* = 6.69 × 10^−6^) with permutation tests shown in Figure S2B. Most of the samples were  assigned to ISUP 2 group (n = 29) or ISUP 3 group (n = 17), while only four samples were assigned to ISUP 1 and five samples to ISUP 4 group, respectively. The score plot of the OPLS-DA model identified two main clusters of samples as seen in Fig. 3A. The first cluster included samples of the ISUP 1 or 2 groups, and the second cluster samples of ISUP 3 or 4 groups. Since the majority of studied samples belonged to ISUP 2 or 3 groups, our further approach was to identify patterns to differentiate solely between those two groups. The OPLS-DA approach generated two main groups reflecting PC aggressiveness: one was the ISUP 2 group and the other the ISUP 3 group as seen in Fig. 3B. This clearly shows, that more aggressive tumors could unambiguously be separated from less aggressive tumors (R2Y = 0.694; Q2 = 0.361; *p* = 8.69 × 10^−4^; a result also confirmed by permutation tests in Additional file 2: Fig. S2C). Figure 4 presents the metabolites which enable the discrimination between tumors of the ISUP 2 group versus the ISUP 3 group. In this figure are also shown the respective levels found in benign samples and in PC samples with different ISUP grades, and the associated results of ANOVA analysis with post hoc Benjamini–Hochberg test. Clearly, the metabolites choline and PC + GPC’s levels increased from benign samples to less-aggressive PC and even further to more-aggressive PC. Even glutamate levels increased with aggressiveness, although the levels in benign samples were significantly higher than in tumor samples with ISUP 1. None of those selected metabolites were significantly correlated to age or levels of serum PSA.

### Three Ki67/PSA based PC subtypes are reflected in metabolomic profiles

Upon classification by Ki67/PSA immunohistochemistry the clinical stages for each subtype are summarized Table 1. The subtype A contains tumors with ISUP grade 1–3, while the subtype B includes tumors with ISUP grade 2–4. The subtype non-AB is characterized by tumors from the ISUP 2 and 3 groups.

**Fig. 5:**
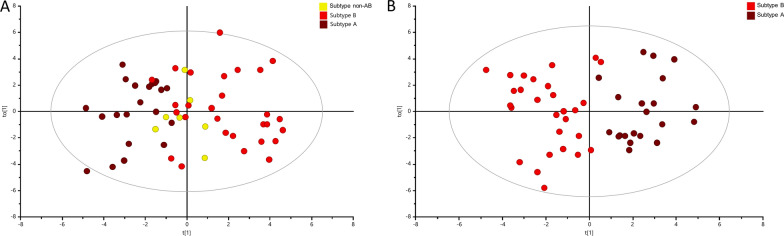
Multivariate analysis obtained from tumor prostate tissues with different PC subtypes. PC subtypes were assigned based on a combinatory Ki67/PSA immunoreactivity score. **A** OPLS-DA score plot showing discrimination between three PC subtypes (brown—PC subtype A; red – PC subtype B; yellow—PC subtype non-AB). **B** OPLS-DA score plot showing discrimination between two main subtypes, A and B (brown—PC subtype A; red—PC subtype B)

**Fig. 6:**
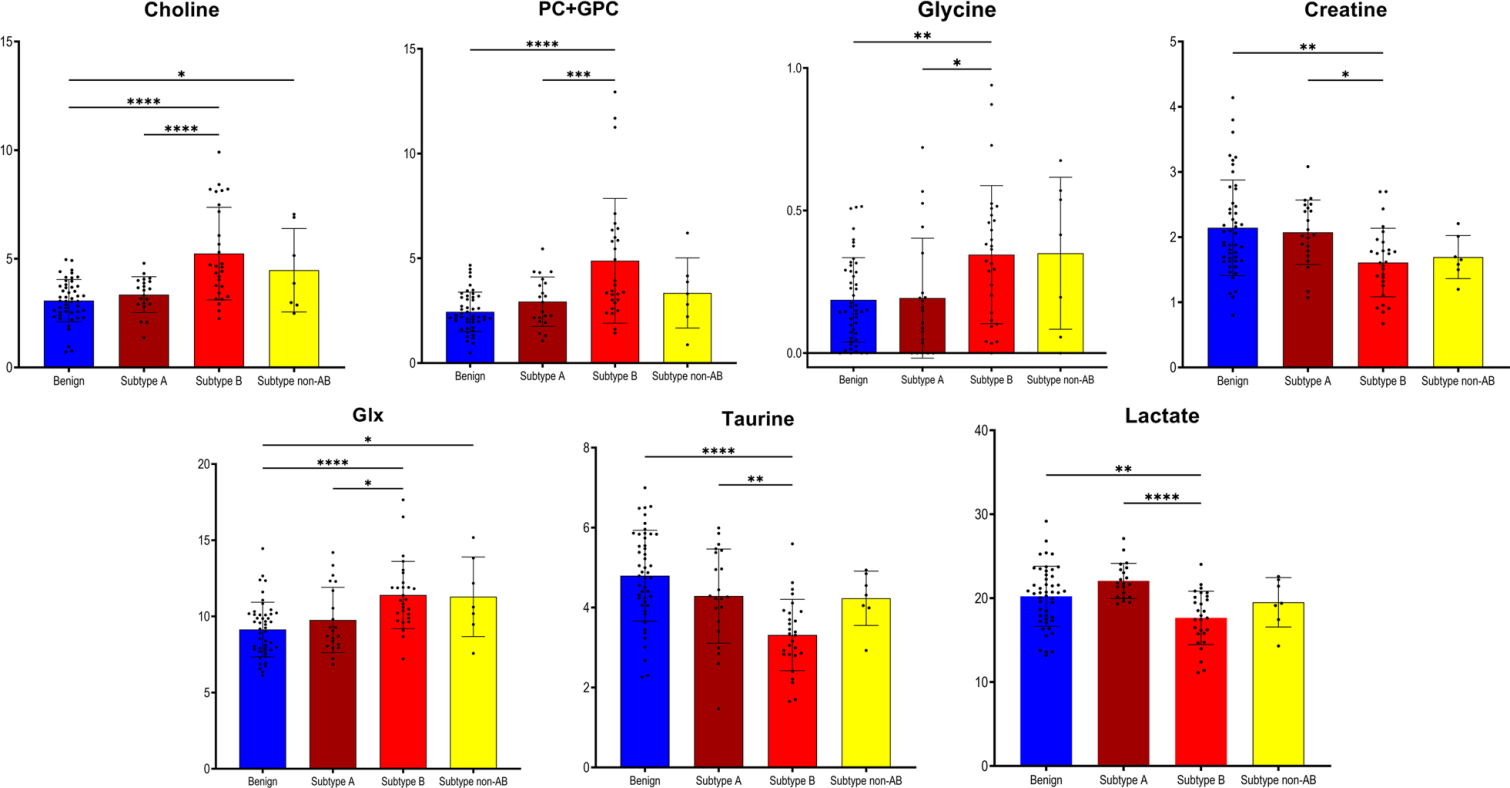
Tissue metabolites as biomarkers to differentiate between subtype A and subtype B. Box plots showing relative abundances for the 7  metabolite-panel to distinguish PC subtypes A and B in addition with data from subtype non-AB and benign prostate tissues. Data are expressed as means ± standard deviation and statistical significance was determined using the one-way ANOVA followed by post-hoc Benjamini-Hochberg (FDR, false discovery rate) test and a *p*-value < 0.05 was considered statistically significant. PC + GPC—peak for combined phosphocholine/glycerophosphocholine metabolites; Glx: combined signal of glutamate/glutamine

Here, we analyzed the distribution of the metabolic patterns in the subtypes A, B and non-AB to address the biological and metabolomic relevance of the Ki67/PSA score-based subtypes. Supervised dimensional reduction analysis, OPLS-DA, of the metabolomic data revealed that those three clinically relevant subtypes of PC had distinct metabolomic profiles, as illustrated in Fig. 5A. Validation of the models indicated their good quality, as seen in the corresponding values for R2Y = 0.375; Q2 = 0.190; *p* = 5.48 × 10^−6^, and the results of permutation test as seen in Additional file 2: Fig. S2D. Especially, subtypes A and B were clearly separated (Fig. 5A), while the non-AB subtype showed a partially overlap and was clustering between those other subtypes. An OPLS-DA analysis was then carried out to identify metabolites that clearly differentiated between the clinically most contrasting A and B subtypes. The corresponding score plot shows a very clear and pronounced separation between the subtypes A and B in Fig. 5B. The “goodness” of the OPLS-DA model was R2Y = 0.745; Q2 = 0.329; *p* = 7.51 × 10^−3^, and the results of permutation tests (Additional file 2: Fig. S2E) showed no signs of over-fitting. Choline, PC + GPC, glycine, creatine, Glx, taurine and lactate were the metabolites found to be significant for differentiation of subtype A versus subtype B. In Fig. 6 their relative levels are compared between the three subtypes of PC and benign samples (ANOVA analysis with post-hoc Benjamini–Hochberg test used here). This analysis highlighted specific pattern of metabolomic profile changes observed between those four groups. Levels of most metabolites in subtype A were found similar to the ones seen in benign samples. Furthermore, there was a more general pattern of either decreased or increased levels of most metabolites from benign samples. This pattern become more pronounced in subtype A and reaching the lowest, respectively highest levels in samples of subtype B. The non-AB subtype displayed intermediate metabolite levels between subtypes A and B.

**Fig. 7:**
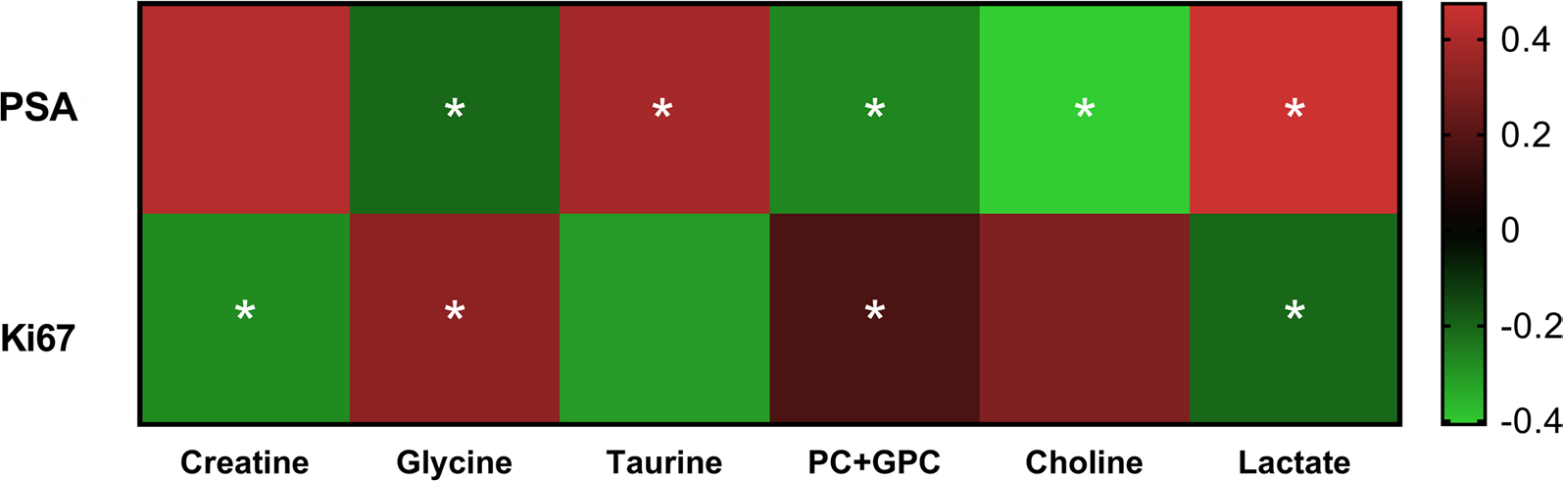
The correlation heatmap between differential metabolites between PC subtypes A and B and PSA and Ki67 values. Levels of Glx did not correlate significantly neither with PSA nor with Ki67. The magnitude of the correlation between the metabolites is shown with red representing a positive correlation and green a negative correlation. *p < 0.05 indicates statistically significant differences

To estimate the correlation strength between the PSA and Ki67 values and individual tissue metabolite levels, Pearson correlations were computed. The corresponding Fig. 7 shows the relevant correlation plots with six out of seven metabolites that differentiated PC subtype A from PC subtype B, also being significantly correlated to PSA and/or Ki67 values. From the panel of those seven selected metabolites, only taurine presented significant but weak positive correlation to age, while no associations with serum PSA levels were observed.

## Discussion

In this study, we used a non-destructing, non-invasive ^1^H HR MAS NMR technique on intact prostate tissues to identify metabolic signatures of PC tissues and variations in those patterns reflecting cancer aggressiveness. Our approach provided an unambiguous differentiation of tumor samples based on metabolite levels related to different processes of clinical and prognostic significance. Our findings could be well correlated to the tumor cell differentiation based on the ISUP grading system and to the combined evaluation of tumor cell proliferation and androgen dependency based on the Ki67/PSA immunoreactive score [14, 15, 27, 34].

We found the predictive value Q2 of the obtained OPLS-DA models to be rather low. Nevertheless, all models were significant as evaluated by CV-ANOVA and permutation tests. It has been shown that in practice it is difficult to give a general limit that corresponds to a good predictability since this depends on the properties of the dataset [35, 36]. Moreover, an acceptable Q2 threshold will depend on the number of observations included [37].

The prostate tissue has a unique metabolic activity which can change severely during tumor development and progress. In contrast to other solid cancers, primary PC does not exhibit a general Warburg effect. Hence, it has been suggested that the ‘‘metabolic switch’’ occurs when PC enters its therapeutic and lethal stages. More precisely, PC exhibits specified metabolic and energetic phenotypes depending on the stage of disease progression as it undergoes two metabolic switches, with the first from anaerobic glycolysis to oxidative phosphorylation (benign glands to primary cancers) and the second towards the lethal metastatic disease state; a state requiring anaerobic glycolytic activity and enhanced fatty acid oxidation [38, 39].

The PC samples in our study showed metabolomic patterns significantly different from benign tissues. Overall, the cohort of PC biopsies displayed common patterns of metabolic adaptation, including an upregulation of glycerophospholipid metabolism. This upregulation was reflected by increased levels of choline, phosphocholine and glycerophosphocholine, a pattern observed by us previously [23, 40]. Even levels of fumarate and succinate were increased with both being metabolites from TCA cycle and suspected to be oncometabolites [41]. In comparison to benign prostate tissues, the metabolome of tumor tissues revealed significant dysregulation of the oxidative pathway, as visible in increased levels of arginine, glutamate and Glx and a decreased level of glutathione (GSH). Similar patterns have also been reported previously [23, 42]. Unfortunately, another useful information, namely the ratio of oxidized form of glutathione (GSSG) and GSH could not be determined, since GSSG could not be clearly distinguished in the NMR spectra. We also observed increased levels of glycine, alanine, glutamate, ascorbate and decreased levels of creatine, myo-inositol and taurine; a behavior correlated with cancerous alterations as suggested previously [42–45].

In the clinical management the Gleason score/ISUP grading system reliably classifies the aggressiveness of a PC tumor and predicts patient outcomes [46]. Here, we applied the new ISUP grading system with Gleason 7 grade tumors being divided into two separate groups. There, ISUP 2 corresponds to Gleason score 3 + 4 and ISUP 3 to Gleason score 4 + 3. Since the ISUP 2 group has a more favourable prognosis than ISUP 3 group [47, 48], our focus here was to identify specific metabolomic differences which even reflect changes in the regulation of biochemical pathways driving PC aggressiveness. We selected six metabolites (choline, PC + GPC, glutamate, Glx, hypoxanthine and methionine) which most prominently reflected the significant metabolomic contrasts between PC with ISUP grade 2 and with ISUP grade 3. The levels for choline and GPCho + PCho metabolites followed a trend of continuously increasing from benign samples through all ISUP grades. Glutamate levels also increased through all ISUP grades, but its levels in benign samples were higher than in ISUP 1. The measured levels for Glx only increased from ISUP 1 to ISUP 3. This behavior of continuous increased/decreased through all ISUP grades was not observed for methionine or hypoxanthine.

Those positive associations between levels of choline/phospholipid metabolites and Gleason score/ISUP grades have been described before [42, 49–51]. Also, an altered phospholipid metabolism has been suggested as a useful tool to exploit for ISUP groups differentiation [43, 52, 53]. High Gleason scores at diagnosis were also seen as a strong predictive factor for positive 18F-choline PET/CT scans in recurrent PC, even when the serum PSA level was low [54]. There, 18F–choline PET scans can even discriminate high-grade prostate lesions from low-grade ones [55].

PC is phenotypically and molecularly very heterogeneous [11, 12], and presents therefore severe challenges for diagnosis and treatment. The unambiguous identification of distinct metabolic signatures for different PC subtypes by us here, provides promising opportunities for diagnostic tools which identify the molecular processes driving PC development to enable a reliable stratification into patient-tailored therapies [16, 56, 57]. With respect to PC heterogeneity, we characterized the metabolic differences between molecular PC subtypes based on the combined Ki67/PSA immunoreactivity score; a useful marker for tumor aggressiveness and providing prognostic information independent to the Gleason and ISUP grading. By using this score, subtype A defines a set of patients where active monitoring could be the preferred treatment [7, 14, 15]. The clear changes seen between the subtype A (Ki67 low/PSA high) tumors and the subtype B (Ki67 high/PSA low) cases are most prominently reflected in tissue levels of seven metabolites. The metabolites choline, PC + GPC, glycine and Glx are significantly higher and creatinine, taurine and lactate are considerably lower in subtype B samples than in subtype A biopsies. The observed metabolomic profile of PC subtype A was similar to benign samples. To differentiate the non-AB subtype was difficult, since its metabolic pattern lay between the patterns found for the A and B subtypes. Not surprisingly, patients with a non-AB subtype are known to have an intermediate prognosis compared to A and B patients [14, 15].

The subtype B cohort contained many samples with a high ISUP Grade. Therefore, higher levels of choline, PC + GPC and Glx observed for subtype B were also reflected in samples with a higher Gleason grade. This behavior reflects an altered choline/phospholipid metabolism. In addition, a higher Gleason grade was associated with increased proliferation as visible by Ki67 marker [40, 58–60]. Interestingly, in more aggressive PCs an increase in phosphocholine metabolites is accompanied by a choline kinase alpha (CHKA) overexpression [61]. This protein is an androgen receptor chaperone which can even be exploited as a marker of tumor progression and as a potential therapeutic target [62].

In addition, the NMR signal for the combined glutamine/glutamate metabolites (Glx) was significantly increased in PC subtype B samples compared to the subtype A samples. Glutamine is critical for cancer cell proliferation and can even be oxidized to glutamate, which can help replenishing the TCA cycle by conversion to α-ketoglutarate [63]. Key regulators of glutamine metabolism include the AR pathway, MYC, and the PTEN/PI3K/mTOR pathway. Apart from the glutamate metabolism, those two metabolites play a role in numerous metabolic pathways. Glutamine is important for cancer growth as a nitrogen donor, in which capacity it supports the increased demand for nucleotide biosynthesis in cancer cells [64]. Therefore, increased glutamate in subtype B may suggest increased proliferation. Moreover, metastatic PC tissues might have an increased glutamine uptake [65]. Also, glutaminase (GLS1), the key enzyme converting glutamine to glutamate, has shown higher expression levels in T3/T4 tumors, and in tumors with higher Gleason scores [66–68]; a behavior suggesting an association of elevated expression with PC progression. Currently, an inhibitor of GLS1, CB-839, is under clinical investigation for a variety of cancer types including PC [69].

In subtype A tissues we found higher levels of lactate and taurine compared to subtype B. Taurine, which is mainly involved in the taurine and the hypotaurine metabolism pathway, can regulate the PI3K/AKT, AKT/FOXO1, JAK2/STAT3 and mTOR/AMPK signal pathways for cell proliferation and protein synthesis. Higher taurine levels have been detected in reactive stroma and suggested to reflect an inflammatory response [70]. However, we also noted a positive correlation of taurine levels with age, which would need detailed exploration in future studies specifically designed for the purpose. Increased levels of lactate indicate an enhanced glycolysis in PC [71, 72], and clinical studies revealed that high-grade PC had significantly increased lactate efflux compared to low-grade PC and benign prostate tissue [73, 74]. Lactate seems to be required for tumor progression [75, 76], and therefore its decreased levels in subtype B compared to A are not really understood yet. Higher levels of creatinine in subtype A found by us confirm previous observations by Patel et al. [77] who suggested an enhanced creatine metabolism as a central component of progressive prostate cancer.

We also detected increased levels of glycine in subtype B compared to A. Glycine itself is an essential amino acid and its increase highlights the relevance of serine/glycine biosynthesis and one-carbon metabolism in cancer development [78]. Furthermore, glycine along with other one-carbon units contributes to the purine and thymidine synthesis which is required for nucleic acid synthesis and cell proliferation [79]. Therefore, the elevated glycine levels also point at an increased proliferation in subtype B. Those increased levels of glycine correlate well with previous studies on more aggressive types of other cancers, like brain cancer [80], breast cancer [81–84] and rectal cancer [79]. It seems that rapidly proliferating cancer cells have increased their glycine-dependence and consumption [85]. Moreover, high cytoplasmic expression of glycine N-methyltransferase (GNMT) have been correlated with a higher Gleason score and higher pT stage, and patients with high GNMT expression showed significantly lower disease-free survival rates compared with patients with low expression GNMT levels [86]. Therefore, increased GNMT and glycine levels may represent novel markers of malignant progression and poor prognosis in prostate cancer.

Taken together, the metabolomics pattern found by us for differentiating PC subtypes A from B reflects the differences in the alterations in a few metabolomics pathways, including increased proliferation as seen in changes in glycine, Glx and even creatinine levels. Moreover, an increased choline/phospholipid metabolism is correlated with higher Gleason score/ISUP grades in subtype B then in subtype A and is reflected by higher levels of choline, phosphocholine and glycerophosphocholine. Additionally, the decrease in taurine levels could suggest an inflammatory response and increased lactate an enhanced glycolysis.

Our classification of PC into three subgroups has many similarities to breast cancer (BC) subtype classification, namely PC subtype A with BC luminal A, PC subtype B with basal-like BC and PC subtype non-AB with BC luminal B [14]. Moestue ﻿et al. [81] compared metabolomic profiles of basal-like and luminal-like breast cancer xenograft models and samples from patients with estrogen/progesterone receptor positive (ER+/PgR+) or triple negative (ER−/PgR−/HER2−) breast cancer. In agreement with our results, they found that more aggressive basal-like breast tumors compared to the less aggressive luminal-like were characterized by significant distinct choline metabolic profile and an increase of glycine. As we, they also observed alteration of taurine and creatine (in our case creatinine), however those changes were not significant. Moreover, their gene expression data suggested a metabolic shift from phosphocholine synthesis to glycine formation in basal-like xenografts, which could also be the case for our data.

Our HR MAS NMR derived tissue-based metabolomics subtypes are a valuable addition to existing prostate cancer molecular classification systems and a powerful resource for understanding the etiology of prostate cancer heterogenicity. Additionally, our study provides information about metabolomic patterns of PC subtypes A, B and non-AB in relation to benign samples. The subtype A is most similar to the noncancer samples on metabolomic level. We propose that those subgroups need different tailor-made treatment, something to be considered by planning novel personalized therapeutic strategies. In future studies, the association between metabolic subtypes reported here and therapy responses might be a powerful tool to refine patient selection for personalized therapy.

In a continuation study, it would be beneficial to more deeply investigate potential therapeutic strategies for this PC subtypes by conducting the targeted detection of the crucial intermediates in those pathways and by utilizing stable isotope tracing experiments to illustrate those pathways in the PC subtypes. It is also important to include higher number of samples to obtain satisfactory prediction values before being able to implement the results in future trials for PC precision medicine.

## Conclusions

In summary, we achieved our long-term goal to elucidate metabolic pathways driving PC heterogeneity into more aggressive disease, information that will provide possibilities for developing subtype-specific treatment strategies. Here, we demonstrated successfully the discriminatory power of non-destructive ^1^H HR MAS NMR spectroscopy on intact biopsy tissues in combination with multivariate statistical analysis for PC subtyping. Our approach allowed unambiguous separation of benign prostate samples from prostate tumors and was able to discriminate PC aggressiveness based on tumor cell differentiation, by separating ISUP grade group 2 from ISUP grade group 3. Most importantly, we could reveal significant differences in the metabolic phenotypes for PC subtypes, previously established based on tumor cell proliferation and androgen dependency, namely, subtypes A, B and non-AB. Specifically, our results from metabolic fingerprinting of intact biopsies have unravelled metabolic characteristics of the highly aggressive PC subtype B (having high proliferation, low androgen dependency, and poor prognosis after conventional therapy), which clearly provides a molecular foundation for the design and implementation of personalized approaches to improve treatment of lethal PC.

### Supplementary Information

**Additional file 1: Table S1.** Metabolites identified by ^1^H HR MAS NMR in prostate tissue samples.

**Additional file 2: Fig. S1.** Analysis of tissue metabolite profiles created for ^1^H HR MAS NMR data. PCA score plot of two groups (blue—benign samples; red—PC samples) with each score representing one subject.

**Additional file 3: Fig. S2.** Plots obtained after performing random permutation test with 200 permutations on OPLS-DA models. **A** Tumor versus adjacent benign prostate tissue. **B** Four ISUP Grade Groups: ISUP 1, ISUP 2, ISUP 3, ISUP 4.** C** IUSP 2 versus ISUP 3. **D** Three subtypes: A, B and non-AB. **E** Subtype A versus subtype B.

## Data Availability

The datasets used or analyzed during the current study are available from the corresponding authors on reasonable request.

## Abbreviations

BC: Breast cancer
CPMG: Carr-Purcell-Meiboom-Gill
Glx: Glutamate/glutamine
GNMT: Glycine *N*-methyltransferase
GSH: Glutathione
HR MAS NMR: High resolution magic angle spinning nuclear magnetic resonance
H&E: Hematoxylin–eosin
ISUP: International Society of Urological Pathology
PC: Prostate cancer
PCA: Principal component analysis
PC + GPC: Phosphocholine/glycerophosphocholine
PSA: Prostate-specific antigen
