# Supplementary figures and images for: Metabolomic profiles of intact tissues reflect clinically relevant prostate cancer subtypes
Source: J Transl Med. 2023 Nov 27;21:860. doi: 10.1186/s12967-023-04747-7 (PMC10683247; doi:10.1186/s12967-023-04747-7)

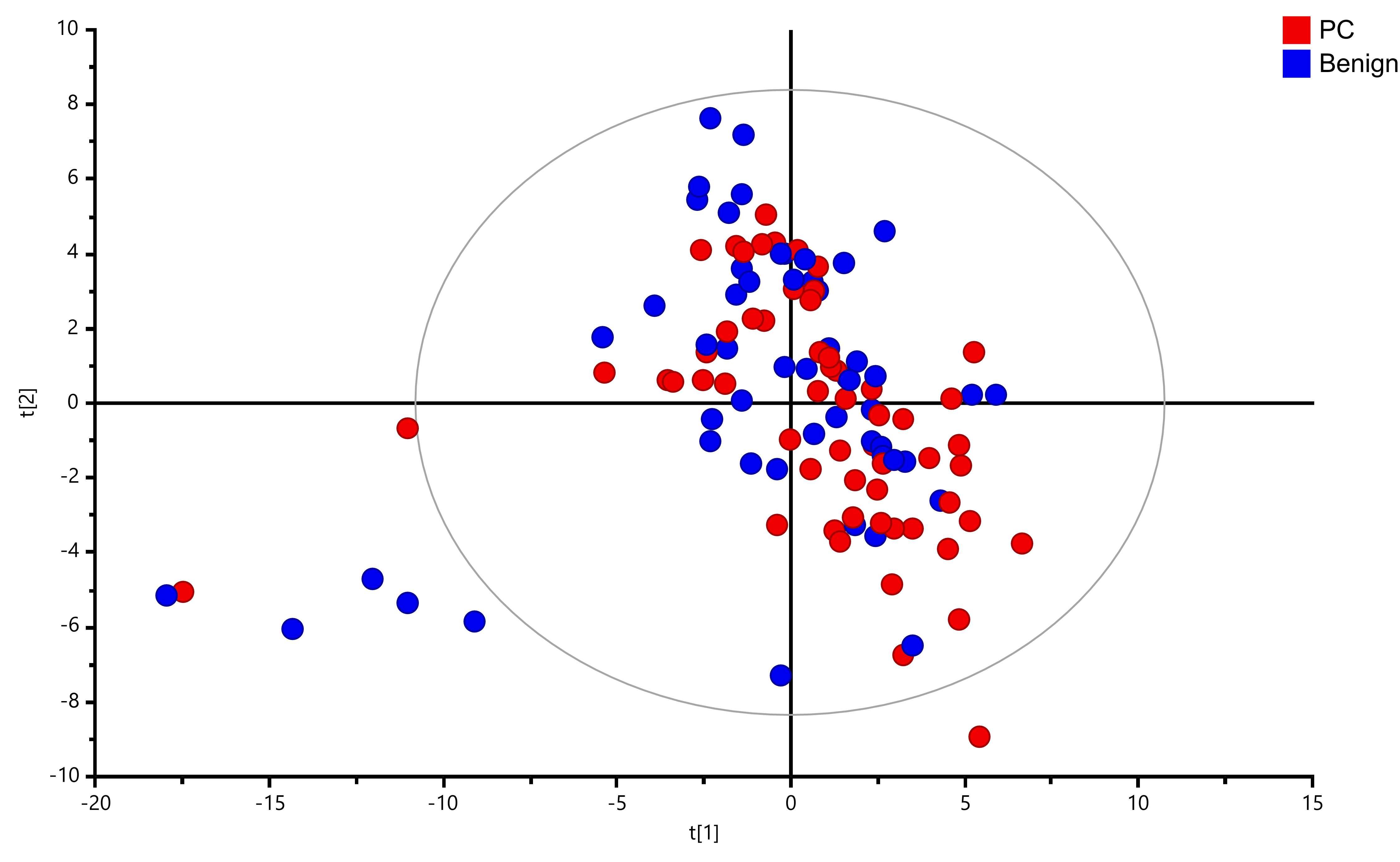

Supplement: Supplementary file 2 — Additional file 2: Fig. S1. Analysis of tissue metabolite profiles created for 1H HR MAS NMR data. PCA score plot of two groups (blue—benign samples; red—PC samples) with each score representing one subject. [file 12967_2023_4747_MOESM2_ESM.tif]

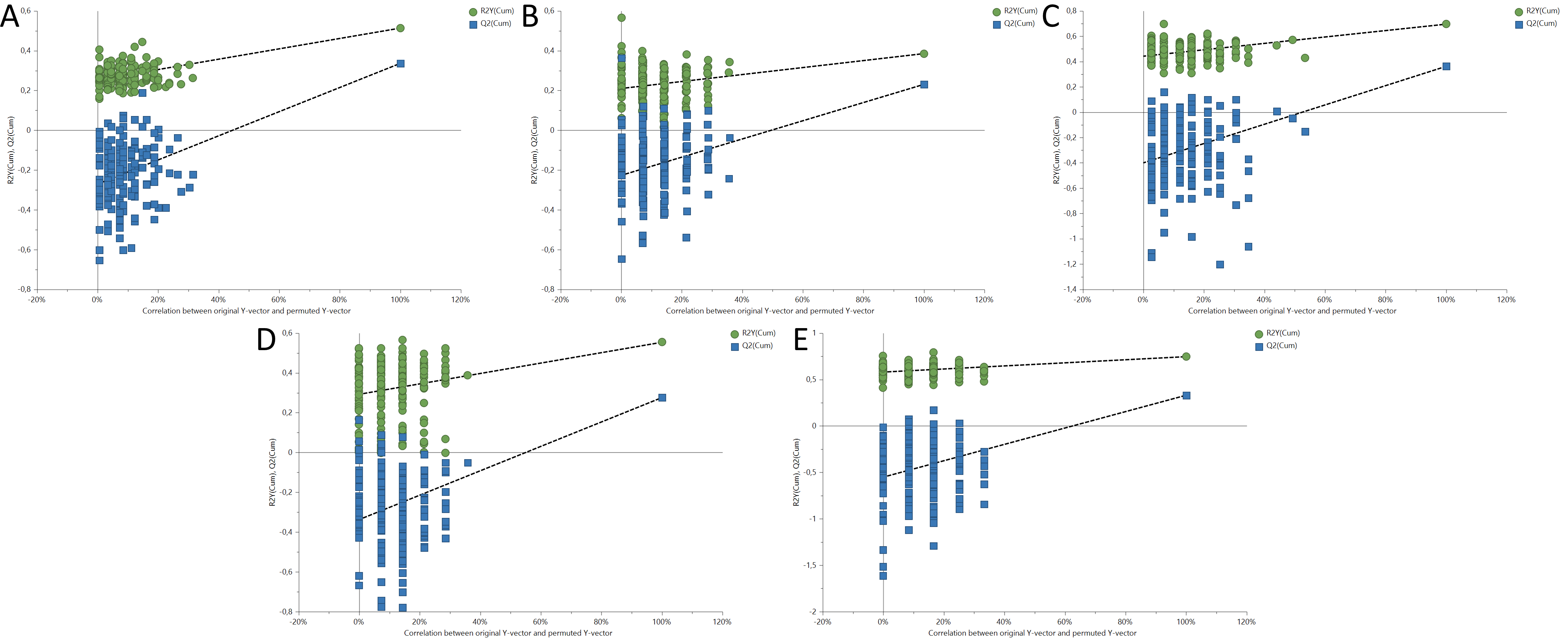

Supplement: Supplementary file 3 — Additional file 3: Fig. S2. Plots obtained after performing random permutation test with 200 permutations on OPLS-DA models. A Tumor versus adjacent benign prostate tissue. B Four ISUP Grade Groups: ISUP 1, ISUP 2, ISUP 3, ISUP 4. C IUSP 2 versus ISUP 3. D Three subtypes: A, B and non-AB. E Subtype A versus subtype B. [file 12967_2023_4747_MOESM3_ESM.tif]
